# Nanofiber Ion-Selective Membrane-Coated Carbon Paper All-Solid-State Sensors: One Stone, Two Birds

**DOI:** 10.1021/acs.analchem.3c04764

**Published:** 2024-02-15

**Authors:** Emilia Stelmach, Justyna Kalisz, Barbara Wagner, Krzysztof Maksymiuk, Agata Michalska

**Affiliations:** Faculty of Chemistry, University of Warsaw, Pasteura 1, 02-093 Warsaw, Poland

## Abstract

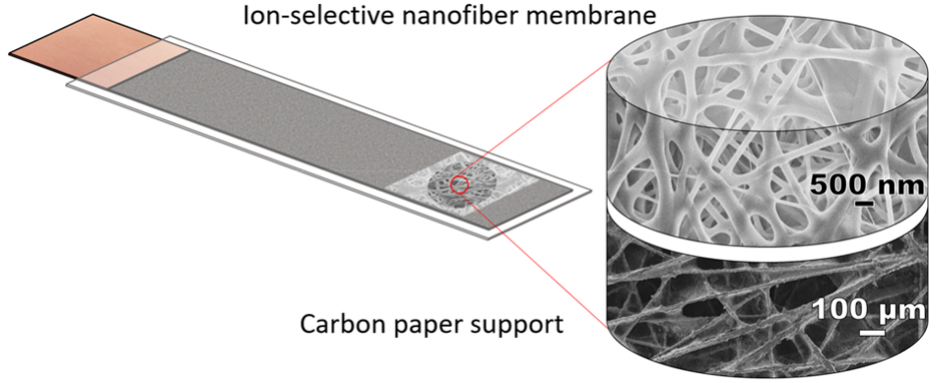

Potentiometric sensors with nanostructural ion-selective membranes were prepared and tested. Electrospun nanofiber mats were applied in novel all-solid-state sensors, using carbon paper as an electronically conducting support. For the sake of simplicity, application of a solid contact layer was avoided, and redox-active impurities naturally present in the carbon paper have proven to be effective as ion-to-electron transducers. Application of a nanostructural ion-selective membrane requires an innovative approach to combine the receptor layer with the support. The nanofiber mat portion was fused with carbon paper in a hot-melt process. Applying temperature close to 120 °C for a short time (3 s) allowed binding the nanostructural ion-selective membrane with carbon paper, without significant changes in the nanofiber structure. This process was conveniently performed together with the lamination of the carbon paper support. The thus obtained, potentially disposable sensors were characterized as exhibiting highly reproducible potential readings in time as well as between sensors belonging to the same batch. The benefits of the application of nanostructural ion-selective membranes include shorter equilibration time, lower detection limit, and significantly lower material consumption. However, the nanostructural membrane is characterized by a higher electrical resistance, which is attributed to higher porosity.

## Introduction

All-solid-state, solid contact ion-selective electrodes (SC-ISE) continuously receive significant research attention.^1,2^ The research is focused mostly on the effect of construction of the sensor,^1,2^ and the ion-selective membranes (ISMs) used are typically continuous films. The final step of SC-ISE preparation is drop casting of a dispersion (cocktail) of polymer, ionophore, and ion-exchanger with an optional plasticizer prepared in adjunctive solvent (followed by solvent evaporation). An alternative approach uses spray coating of the membrane dispersion, resulting in continuous film formation in a way that eliminates potential human error factors; however, it is still experimentally quite complicated.^3^

Although much research has been devoted to the effect of the composition of ISMs on sensor performance, especially in the early days of potentiometry, it is surprising that other methods of ISM application, such as alternative membrane formats, have not been considered. To the best of our knowledge, ISM is typically understood as a continuous layer (film), and application of other membrane formats and the potential benefits of nanostructural ion-selective membranes have not been explored for SC-ISEs. However, the results obtained for internal-solution ISEs or optodes are encouraging.^4,5^ The polyvinylidene fluoride (PVDF) nanofiber mat surface modified with plasticizer containing ionophore and ion-exchanger used as ion-selective membrane in internal solution ISEs was characterized with an analytical performance equivalent to those of classical systems, yet it offered short conditioning time and under electrochemical trigger behaves similarly to an array of nanoelectrodes.^4^ The aim of this work was to investigate the possibility of application of a nanostructural ISM in SC-ISEs. One of the challenges is finding an efficient way to bind the nanofiber ISM with the support of choice.

The significant advantage of the SC-ISE concept is the simple construction, allowing (potentially) disposable use and reducing maintenance, paving the way to calibration-free sensors.^1,2^ The first proposed, ultrasimple construction of metal coated with an ion-selective membrane, i.e., coated wire electrode,^6−8^ did not offer sufficient stability of potential readings in time due to ill-defined ion and charge transfer at the interface between the ISM and the electronically conducting support, i.e. a blocked interface.^9^ To overcome this problem, the simplicity of construction needs to be compromised, and an additional layer has to be placed between the ionic conductor (ISM) and electronic conductor (support)—an ion-to-electron transducer. The transducers proposed range from conducting polymer-doped (oxidized) as well as semiconducting ones, carbon nanostructural materials, to metallic nanostructures and many others systems (for examples, see refs (1, 2, and 10)).

Recent studies show that in fact good reproducibility of SC-ISEs’ potential reading in time is achieved even if the system applied as transducer is of relatively small redox capacity.^11−13^ Surprisingly, even systems composed (nominally) of just one redox reagent (not a redox pair) offer good stability and reproducibility of potentials readings in time; this effect was explained by the presence of redox-active impurities in applied reagents and mixed potential formation resulting from spontaneous processes of the present redox reactants.^11−13^

On the other hand, many (if not all) transducers proposed, including most promising ones, have system-specific drawbacks, e.g., high primary/interfering ion content^12^ or tendency to partition into the membrane phase^14^ (both potentially compromising analytical performance). Highly promising hydrophobic conducting polymers^10,15^ require electrochemical polymerization of the transducer from an organic solvent.

In order to develop disposable sensors, replacement of metal or glassy carbon supporting electrodes by other systems is needed. Toward this end, screen-printed electrodes, e.g., foil or paper modified (painted, spray coated) with conducting polymer and/or carbon nanostructures,^11,16−18^ graphene paper,^19,20^ and 3D drawn/printed polylactide–carbon composites,^21^ have been proposed. These systems require the effective insulation of the conducting track from the sample solution. Among other approaches, lamination using office laminating device and materials was found to be effective; however, this still required application of an ion-to-electron transducer.^19,20,22^

In this work we explore the possibility of making ISEs by merging two elements: ion-selective nanofibers as membrane and supporting electrode in the hot-melt process, performed using an office laminator. This approach allows the application of a nanostructural ion-selective membrane in SC-ISEs for the first time. The new approach allows also avoiding application of transducer layer, a necessary step in all systems proposed so far (except for coated wire type sensors).^9−22^

In order to prepare reliable potentiometric sensors, we aim to study the possibility of benefiting from the presence of impurities in a commercially available conducting material applied as ion-to-electron transducer. The proposed system using carbon paper as support, by analogy to coated-wire type ISEs, was called a coated-carbon paper sensor. Ion-selective nanofiber mat potassium membranes were prepared and tested.

## Experimental Section

Carbon paper (AvCarb GDS 1120, 184 μm) was purchased from Fuel Cell Store. Foil for the laminators, 100 μm, was obtained from Office Supplies International.

### Preparation of the Sensors

ISE nanofiber mats were prepared using the electrospinning method. The classical composition of K^+^-selective membrane was used.^9^ First, a suspension of PVC (601 mg) with DOS (1313 mg) was dissolved in 3 mL of a mixture of THF and DMF in a ratio of 1:1 (v/v) with a magnetic stirrer and heated to 60 °C for 24 h. If not stated otherwise, separately 55.1 mg of valinomycin and 26.2 mg of KTFPB were dissolved in 0.7 mL of solution of THF/DMF (1:1 v/v). Then both solutions were mixed with a magnetic stirrer (30 min) and loaded into the syringe. For comparison in some experiments, nanofiber mats were prepared from a similar solution but containing NaTFPB instead of KTFPB.

The electrospinning apparatus consisted of a DC power source (ELSR30P300, Technix). The obtained mat area was close to 350 cm^2^, allowing preparation of ca. 550 individual sensors. After electrospinning, the ISE nanofiber mats were left in the laboratory atmosphere overnight; after that the mat was stored in a refrigerator (4 °C).

**Figure 1:**
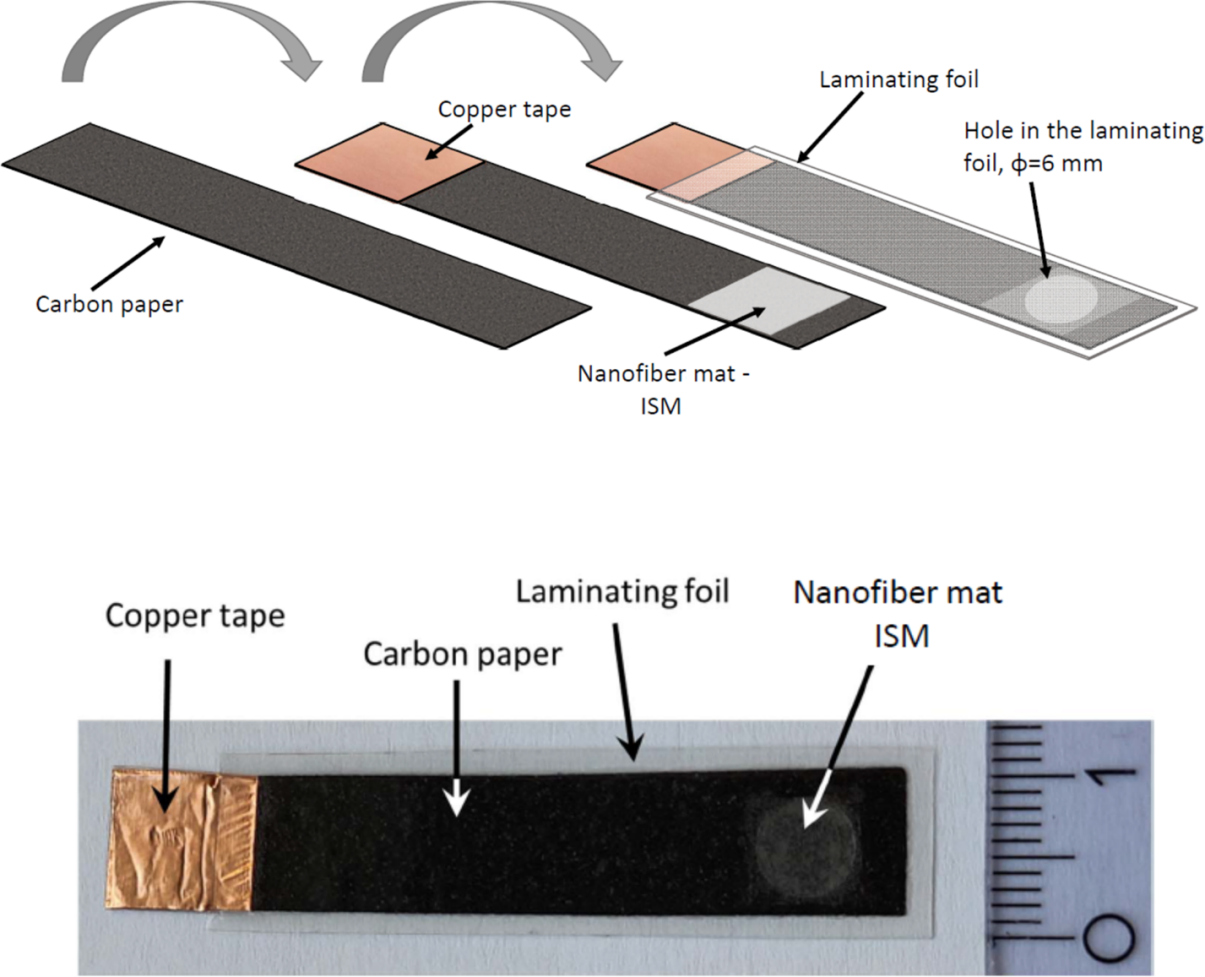
Schematic representation of sensor assmebly and a photo of an obtained SC-ISE with a fiber mat with a scale bar (1 cm).

A 5 × 0.9 cm^2^ rectangle was cut from carbon fiber paper (carbon fiber paper PTFE treated, AvCarb Material Solutions) and used as a support for receptor layers. A 5.3 × 1.2 cm^2^ rectangle was cut from laminating foil. One circular hole with a diameter of 6 mm was made with a hole puncher ca. 6 mm in the upper part of the foil. Square 0.8 cm × 0.8 cm nanofiber mats were carefully placed on carbon fiber paper (Figure 1) below the hole made in the upper part of laminating sheet, making sure that the nanofiber mat stretched further than circular opening in the laminating foil. The whole system was fused using a hot-melt process; this was accomplished by feeding it into the laminating device. Thus, the nanofiber mat was bound with the support and the whole system was laminated–insulated from the solution. The laminator used (Fellowes, Saturn 3i A4) had a working temperature of ca. 120 °C; sensor contact time with the hot rollers was ca. 3 s.

The carbon paper sheets were uncoated only at one end (an electrical output) with copper tape (before laminating) to secure it mechanically for connecting many times.

The thickness of the resulting membranes was equal to 40 ± 5 μm (*n* = 5), and the mass of nanofiber membrane was 1.5 mg. If not otherwise stated, the membranes (mats and continuous films) were prepared by using KTFPB.

Preparation of classical membranes K-ISE is described in the SI. The thickness of resulting membranes was equal to 120 ± 10 μm (*n* = 5), and the mass of the membrane was 3 mg.

If not stated otherwise, the obtained sensors (either with mats or continuous film membranes) were preconditioned before experiments for 40 min in 10^–3^ M KCl.

## Results and Discussion

The motivation for this work was to propose an all-solid-state potentiometric sensor with an ion-selective membrane of truly nanostructural character, competitive performance, and simplified construction, allowing the assembly of sensors from parts.

For the sake of simplicity, we aimed at application of ISM directly on the supporting electrode, eliminating the transducer application step. In line with previous reports,^10−13^ the preference was given to systems containing (naturally) redox-active impurities that are relatively hydrophobic. The applicability of commercially available carbon paper as a support/conducting track was tested. It is not uncommon that carbon materials contain transition metals impurities;^23^ it can be expected that these constituents are present in commercial carbon paper.

### Supporting Electrode

As-obtained carbon paper was characterized with water contact angle 131° ± 0.9° (Figure S1), proving the high hydrophobicity of this material. Surface modification by drop casting DOS resulted in better wettability of the material, yet the contact angle was still relatively high and equal to 110° ± 0.7° (Figure S1).

The cyclic voltammogram recorded for carbon paper in 0.1 M KCl (Figure S2) clearly proves the redox activity of this material, which is enhanced after modification with a minute amount of DOS plasticizer to increase wettability (Figure S2).

**Figure 2:**
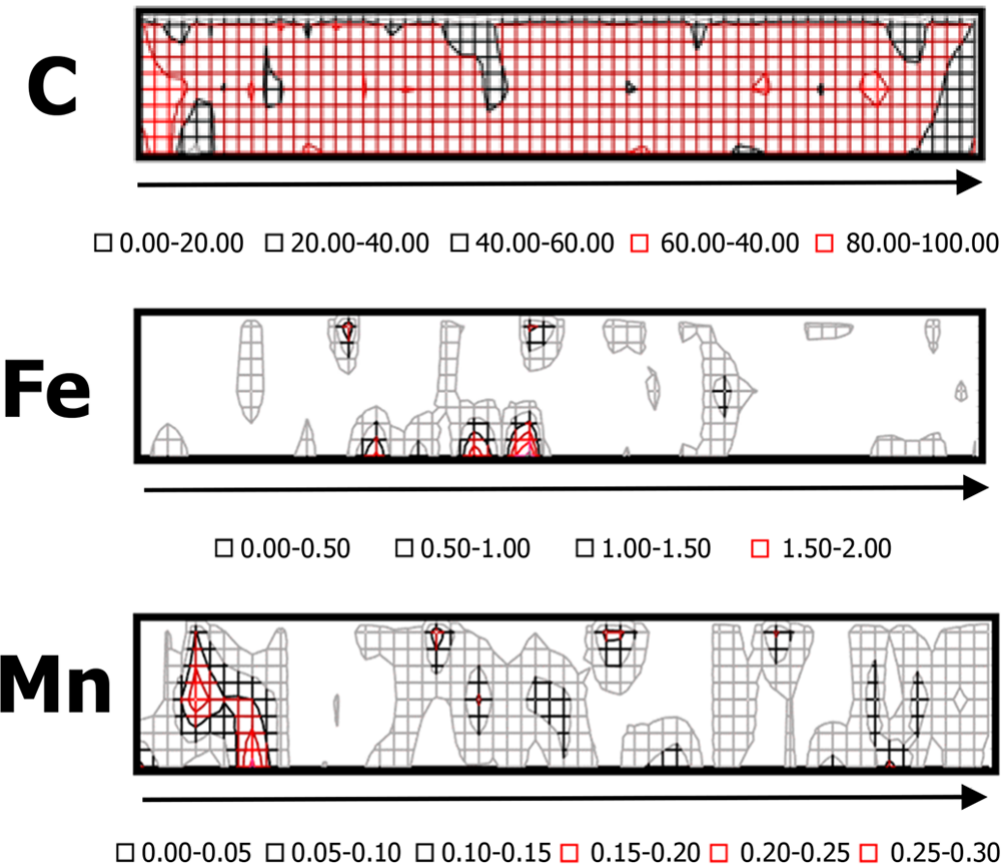
LA-ICP-MS imaging analysis of carbon paper and amount of elements in atomic %.

The elemental composition of carbon paper studied, using a LA-ICP-MS approach, shows that the material contains mostly carbon atoms (ca. 80–100% of atoms), but also iron and manganese atoms in the levels close to 2 and 0.3 atomic %, respectively (Figure 2). Impurities are randomly located in the paper, forming small clusters. Moreover, also other impurities can be found in the paper, e.g. calcium (up to 40 atomic % in spots), as well as lower amounts of copper, zinc, mercury, and lead (up to 0.1 atomic % in spots). Although the chemical nature of these metallic impurities is unknown, due to their properties (different oxidation states are available for, e.g., iron or manganese), these are likely to ensure a stable redox potential at the back side of the membrane. Thus, application of carbon paper (with its impurities) can be an alternative method, allowing preparation of no-added-transducer sensors, i.e., coated carbon paper type sensors. These systems containing redox constituents are expected to be free from the drawbacks typical for coated-wire type sensors.

### Ion-Selective Nanofiber Membrane and Fusing It with Carbon Paper Support

**Figure 3:**
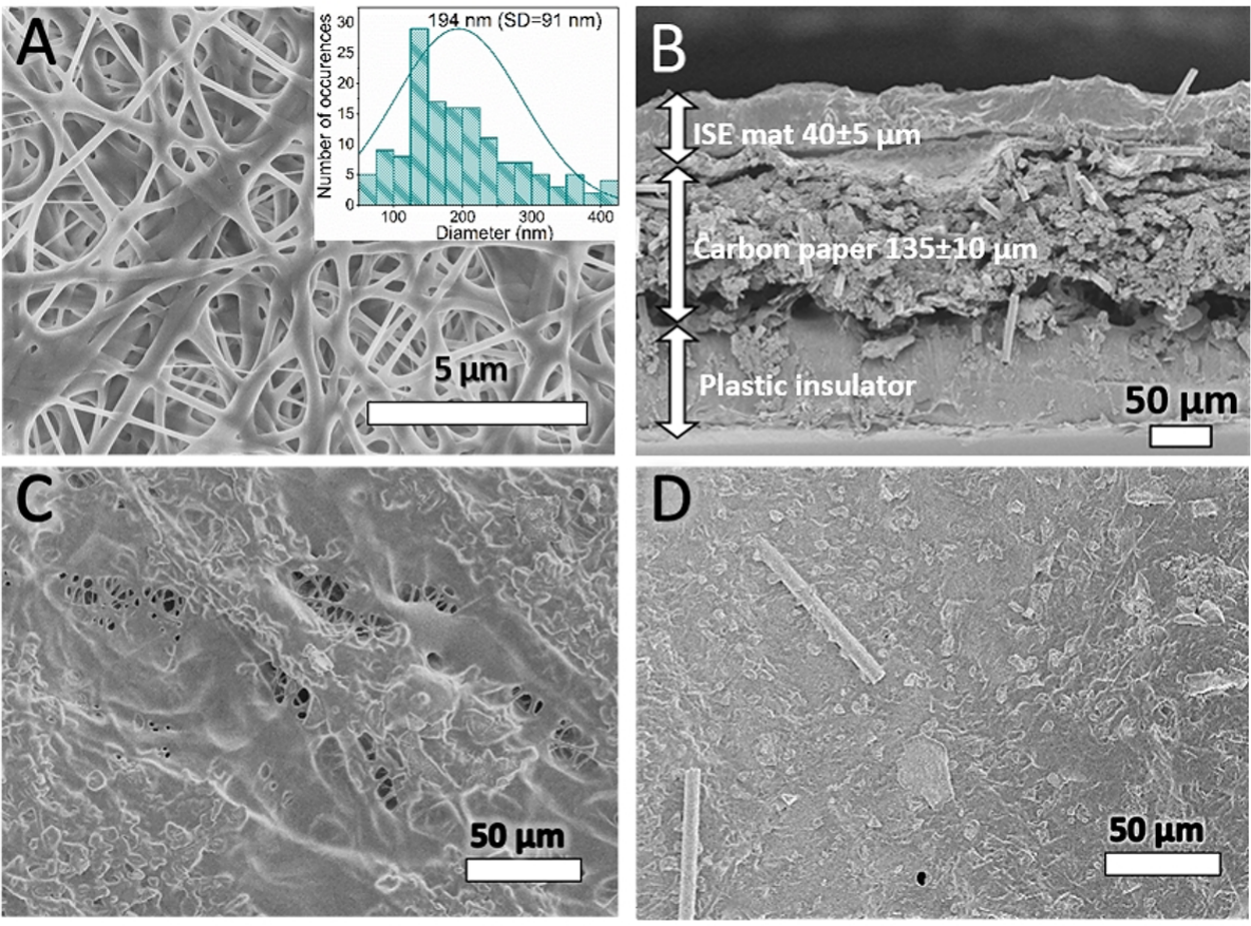
(A) SEM images of nanofiber ISM (top view); inset: size distribution of obtained fibers. (B) Cross section of a laminated electrode with nanofiber ISM. (C) SEM images of nanofiber ISM (top view) post hot melt process, (D) SEM image of back side of nanofiber ISM peeled from the carbon paper.

We report here for the first time the electrospinning of ion-selective nanofibers, i.e., plasticized PVC containing an ionophore and ion-exchanger. The obtained mat (Figure 3A) contains nanofibers of different diameters; however, the majority of these have the diameter between 100 and 300 nm (mean 194 ± 91 nm, Figure 3A). The as-prepared nanofiber mat, ion-selective membrane, has a porous structure, which is typical for this class of nanostructural materials.

To assembly the sensor, ion-selective nanofibers need to be merged with the supporting electrode to ensure electrical contact. To achieve this, we intended to apply the hot-melt approach, to partially transform the membrane to liquid (melt) yet generally preserving its nanostructural character. To the best of our knowledge, this approach has not been explored for an ISM application before. From the practical point of view, it seems attractive to couple this process with lamination of the support carbon paper electrode, leading to easy assembly of the whole sensor.

The effect of temperature on the plasticized PVC nanofibers was studied using a microscope and heating plate. Starting from temperatures close to 80 °C some changes in the nanofibers were observed; initially, these are only local and relatively small (Figure S3). When the temperature was increased to ca. 120 °C, part of the nanofibers was melted; however, it should be stressed that the overall nanostructural character of the mat was preserved (Figure S3). At temperatures close to 170 °C, the mat was fully transformed into a liquid.

Partial melting of the mat is essential to achieve good adhesion of nanostructural ISM to the support (to fuse the parts of the sensor); moreover, local (partial) melting of the mat is expected to cover (insulate) the supporting electrode below the nanofibers.^24^ The process was performed in a laminating device using 120 °C for 3 s; in addition, the mechanical stress of the hot rollers seems to be a good compromise between achieving adhesion of the ISM to the support and preserving the intrinsic nanostructural character of the nanofibers.

Although some of fibers are partially flattened and merged after the hot melt process, clearly the nanostructural character of the ISM is preserved, especially in the deeper layers (Figure 3C). Figure 3B also shows SEM image of the cross section of the prepared sensor. The picture clearly shows that the mat and support are fused together. The SEM image of the back side of the nanofiber ISM (i.e., the side that was in contact with carbon paper, Figure 3D) after mechanically removing it from the support clearly shows carbon structures attached to the mat (Figure S4). Thus, the hot-melt process allows effective merging of the nanofiber mat and the support.

### Nanofiber Ion-Selective Membrane-Coated Carbon Paper All-Solid-State Sensors

The water contact angle determined for nanofiber ISM after the hot-melt process was close to 88° (compared to 89° before hot-melt process) (Figure S5A); thus, it was comparable with previously reported values for a polyvinylidene fluoride (PVDF) system surface modified with DOS.^4^ The water contact angle determined for classical (continuous film) membrane was equal to 74° (the same value was obtained before the hot-melt process) (Figure S5B). This clearly shows that the hot-melt process does not alter the wettability of the ISM, regardless of its type.

It should be stressed that the nanofiber ISM was close to 3 times thinner compared to the classical membrane, and a thinner membrane is expected to equilibrate with a solution in a shorter time. Moreover, due to the high surface area-to-volume ratio of individual fibers for this type of system, equilibration with solution is expected to be faster than for bulk films (of equivalent thickness). For a system containing primary ions added to the membrane composition as ion-exchanger counterions, as studied here, the equilibration process is mainly related to hydration of the membrane.^25^

As expected (Figure S6), the potential values of nanofiber ISM sensors stabilize after ca. 1 h, i.e., faster compared to film membrane type sensors (e.g., see ref (19)). This effect is important for, e.g., simplification/shortening of the pretreatment procedure.

**Figure 4:**
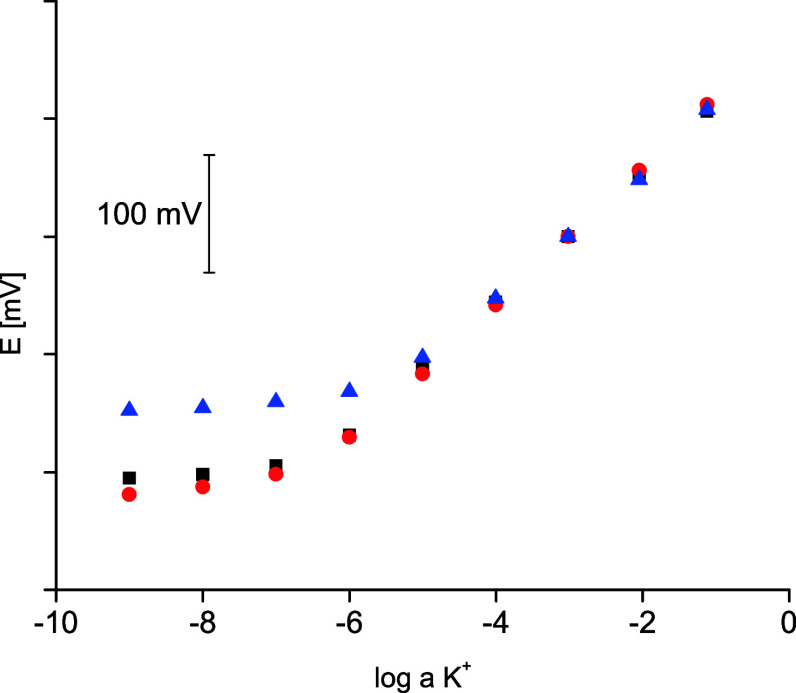
Potentiometric responses of carbon-coated potassium selective electrodes with hot-melt process nanofiber ISM (black squares) after short conditioning (40 min) or (red circles) after conditioning for 20 h in 10^–3^ M KCl and for (blue triangles) typical hot-melt film membrane after short conditioning (40 min), recorded in KCl solutions.

As can be seen in Figure 4, sensors with nanofiber ISM hot-melt fused to carbon paper were characterized with linear potential dependence on logarithm of potassium cation activity within the activity range of 10^–1^ to 10^–6^ M with a slope close to Nernstian and equal to 56.3 ± 0.2 (*R*^2^ = 0.999); the detection limit obtained was equal to 10^–6.6^ M (Table S1). Application of a classical conditioning procedure, 20 h of contact of nanofiber ISM sensor, did not affect significantly the observed responses: the linear response range covers activities from 10^–1^ to 10^–6^ M, the slope of the dependence was equal to 58.1 ± 0.5 (*R*^2^ = 0.999), and the detection limit obtained was equal to 10^–6.7^ M. Thus, the performance of this type of sensor was similar to that of membranes placed on laminated support^19^ or electrodes with spray coated, porous, ion-selective membranes.^3^ It should be added that the tested parallel film (classical) membrane sensor was characterized by somewhat less favorable performance: shorter linear response range, detection limit equal to 10^–5.8^ M, close to Nernstian slope equal to 53.7 ± 1.4 (*R*^2^ = 0.998) (Figure 4).

Sensors prepared using NaTFPB ion-exchanger, both nanofibers and film ISMs, after short pretreatment showed super-Nernstian dependence (Figure S7). However, the magnitude of this effect was significantly smaller for nanofibers used as ISM, due to the significantly lower flux of potassium ions. Moreover, for a sensor with a nanofiber-based ISM of reduced thickness (20 μm thick mat instead of 40 μm one), the effect of a super-Nernstian slope was not observed after short conditioning (Figure S7).

The selectivity coefficients calculated for nanofiber ISM-based sensors as well as classical film ISMs tested in parallel are shown in Table S1. As can be seen from Table S1, regardless of ISM type used, similar values of selectivity coefficients within the range of experimental error were obtained.

Thus, the above presented results clearly show that a nanofiber mat-based ISM coated on carbon paper can be applied using a significantly shortened conditioning procedure and yet offers competitive analytical performance.

The within-day reproducibility of nanofiber ISM-based carbon-coated sensors, expressed as SD of mean potential recorded for a given concentration from 8 sequential calibrations, did not exceed 1.7 mV (for experiments performed in an open beaker, without extra housing, etc.) for the concentration range from 0.1 to 10^–5^ M (Figure S8A). The obtained value is highly promising especially as the ion-to-electron transduction was based on the presence of impurities in the carbon paper used. Slightly higher SD values were obtained for concentrations ranging from 10^–6^ M to 10^–9^ M (ranging from 2.1 to 4.1 mV). This effect is, however, as expected and as typically observed for low concentration ranges.

The within-batch reproducibility of prepared sensors, expressed as SD of mean potential recorded for a given concentration for 10 individual sensors prepared in one batch, did not exceed 2.5 mV for all concentrations tested from 0.1 to 10^–9^ M (Figure S8B). Although higher values of sensor reproducibility have been reported, these were obtained for other systems using applied transducers, e.g., gold-coated glassy carbon disk with modified redox buffer^26^ or nanostructured carbon materials on glassy carbon,^27^ making comparison with the simplified construction difficult.

To further prove the effectiveness of the lamination approach, the effects of the solution redox couple on recorded potentials were tested. As can be seen in Figure S9, potentiometric responses recorded in the presence of redox buffer in solution were not affected by the change of the redox reactant concentrations. The potentials recorded in the presence and absence of O_2_ in the solution were also similar within the range of experimental error, proving that oxygen does not affect the measured potentials.

The water layer test performed^28^ for nanofiber mat ISM sensors (Figure S10) did not indicate formation of a water layer within the sensors, between the nanofiber mat and support–carbon paper, after lamination.

Electrochemical impedance spectra (EIS) of nanofiber sensors and those with classical (continuous) film ISM are shown in Figure S11A. According to the equivalent circuit shown in Figure S11B, the impedance spectra of nanofiber ISM-based sensors present a high-frequency semicircle, suggesting a membrane resistance close to *R* = 7 × 10^5^ Ω and parallel connected resistance (geometric resistance of the membrane) close to *C*_g_ = 2 × 10^–10^ F, both for the typical film membrane and for the mat. The linear dependence shown for lower frequencies represents a constant phase element (CPE) behavior related to carbon paper–solid contact. The admittance related to CPE for the frequency of 1 Hz is close to 4 × 10^–7^ Ω^–1^ and 1 × 10^–6^ Ω^–1^ for the mat and the film membrane, respectively. The factor *n* representing the constant phase (−90·*n* degrees) are 0.65 and 0.53 for the mat and the film membrane, respectively. The obtained resistance of the nanofiber ISM sensor is well comparable with that of classical (continuous) membranes applied on a support coated with a transducer of choice (e.g., see ref (9)). The resistance of the tested sensors, estimated from chronopotentiometric experiments performed in 0.1 M KCl using a cathodic/anodic current equal to 10^–8^ A, was close to 7 × 10^5^ Ω, both for nanofiber ISM and continuous film membrane (Figure S11B). These values are consistent with those obtained from EIS measurements. This experiment shows also close to linear dependence of the potential on time with a small curvature, pointing to diffusion limitations across the membrane resulting from transport of either ions or ionophores. These limitations related to lower mobility are more exposed (higher slope of potential vs time dependence) for the nanofiber ISM sensor. This result is in agreement with the results presented in Figure S7, showing lower super-Nernstian effect and thus lower potassium ion mobility for the electrode with nanofiber ISM, compared to the sensor with film membrane. The lower mobility in the nanofiber membrane may be explained by higher hydrophobicity (as shown by contact angle measurements, Figure S5), making wetting of the structure more difficult.

## Conclusion

In this work, we propose potentiometric sensors prepared using a hot-melt/lamination process to fuse a nanostructural ISM (nanofiber mat) and carbon paper to result in highly reproducible sensors. The hot-melt process does not affect the intrinsic nanostructural character of the membrane, yet it allows the binding of the ISM and the support. Although the sensors proposed herein do not use an additional layer of ion-to-electron transducer, the redox-active impurities present in the carbon paper assure stable redox potential at the back side of the membrane. The nanostructural ion-selective membrane results in a lower detection limit and a faster potential stabilization compared to classical (continuous film) parts.

## Supporting Information Available

Experimental details for apparatus, reagents used, EIS and chronopotentiometric experiments, SEM, LA-ICP-MS mapping, water contact angle determination, electrospinning procedure, and water contact angle determined for unmodified carbon paper support and carbon paper modified with DOS; cyclic voltammograms of DOS modified and unmodified carbon paper; images of changes of the nanofiber membrane structure during heating; images of nanofiber mat, carbon paper, and nanofibers ISM removed from the structure of hot-melt fused sensor; water contact angle determined for nanofiber ISM before and after hot-melt process; change of potential of tested sensors during equilibration with KCl solution; calibration line parameters; detection limits and selectivity coefficients; potentiometric responses of sensors prepared using NaTFPB ion-exchanger; reproducibility of prepared sensors; effect of redox reactants present in the solution on potentials recorded for nanofiber ISM-based sensors; water layer test results; chronopotentiometry and electrochemical impedance spectroscopy results (PDF)
