# Supplementary material for: Nanofiber Ion-Selective Membrane-Coated Carbon Paper All-Solid-State Sensors: One Stone, Two Birds
Source: Anal Chem. 2024 Feb 15;96(8):3253–8. doi: 10.1021/acs.analchem.3c04764 (PMC10902807; doi:10.1021/acs.analchem.3c04764)
Supplement: Supplementary file 1 — ac3c04764_si_001.pdf [file ac3c04764_si_001.pdf]

Supporting Information

Nanofibers ion-selective membranes coated carbon paper all-solid-state sensors - one stone two birds

Emilia Stelmach†, Justyna Kalisz†, Barbara Wagner, Krzysztof Maksymiuk,

Agata Michalska*

Faculty of Chemistry, University of Warsaw, Pasteura 1, 02-093 Warsaw, Poland

* agatam@chem.uw.edu.pl, +48 22 55 22 331

Table of contents

1. Experimental details for apparatus, reagents used, EIS and chronopotentiometric experiments, SEM, La-ICP-MS mapping, water contact angle determination, electrospinning procedure.

2. Water contact angle determined for unmodified carbon paper support and carbon paper modified with DOS.

3. Cyclic voltammograms of DOS modified and unmodified carbon paper.

4. Images of changes of the nanofibers membrane structure during heating.

5. Images of nanofibers mat carbon paper and nanofibers ISM removed from the structure of hot-melt fused sensor.

6. Water contact angle determined for nanofibers ISM before and after hot-melt process.

7. Change of potential of tested sensors during equilibration with KCl solution

8. Reproducibility of prepared sensors.

9. Effect of redox reactants present in the solution on potentials recorded for nanofibers ISM based sensors.

10. Water layer test results.

11. Electrochemical impedance spectroscopy and chronopotentiometry results.

12. Calibration lines parameters, detection limits and selectivity coefficients, potentiometric responses of sensors prepared using NaTFPB ion-exchanger.

Experimental

Apparatus

In the potentiometric experiments and electrochemical measurements experimental setup was used including Lawson Labs. Inc. (3217 Phoenixville Pike, Malvern, PA 19355, USA), the pumps systems 700 Dosino and 711 Liquino (Metrohm, Herisau,) and galvanostat-potentiostat CH-Instruments model 660A (Austin, TX, USA). A double junction Ag/AgCl reference electrode with 1 M lithium acetate in outer sleeve (Möller Glasbläserei, Zürich, Switzerland) was used. The recorded potential values were corrected for the liquid junction potential calculated according to Henderson approximation.

To obtain SEM images FE-SEM Merlin (Carl Zeiss) apparatus was used with SE2 type detector.

The melting point of polymeric mat or membrane was recorded with the Boetius apparatus (VEB Analytik Dresden, HMK 73/4437, Germany), employed with a thermometer, optical microscope, and digital camera (Levenhuk, Inc., USA). The heating rate was about 4 °C/min. The sample was placed between two microscopic glass plates on the heating table during measurements. (VEB Analytik Dresden HMK 73/4437).

Reagents

Valinomycin, potassium tetraphenylborate (KTFPB), sodium tetraphenylborate (NaTFPB), poly(vinyl chloride) (PVC, high molecular weight), bis(2-ethylhexyl) sebacate (DOS) and tetrahydrofuran (THF) were from Aldrich(Germany). All salts used were of analytical grade and were obtained from POCh (Gliwice, Poland).

Doubly distilled and freshly deionized water (resistance 18.2 MΩ cm, Milli-Qplus, Millipore, Austria) was used throughout this work.

Electrochemical experiments

AC impedance spectra were collected over a wide frequency range (0.01−100 000 Hz) at potential equal to 0.3 V and amplitude of 50 mV. The data were collected in 0.1 M KCl solutions.

Chronopotentiometric measurement were conducted using cathodic / anodic current $1 \cdot 10^{-8}$ A in 0.1 M KCl solution.

SEM

To obtain SEM images samples of plasticized polymer were coated with Au/Pd using an Emitech sputter coater for 15 s, and then observed in secondary electron mode. The morphology of the samples was analyzed using SEM with accelerating voltage of 3 kV.

Water contact angle

Water contact angle measurements were carried out with Delta Optical Smart 5MP PRO digital microscope and Fiji / Image J program using 2 μL water drop. The measurement of the contact angle was repeated 6 times, the obtained angle values were averaged and SD was calculated.

Laser ablation inductively coupled mass spectrometry

The laser ablation system LSX-213 (Teledyne CETAC, USA) coupled to a quadrupole mass spectrometer NexION300D (Perkin Elmer, Germany) was used for LA−ICP−MS measurements. The samples were placed horizontally on an X−Y−Z-translation stage inside the ablation chamber, which was continuously flushing with Ar. The exact position of the samples was observed with a CCD camera under PC control. The applied laser energy was 3.2 mJ, the repetition rate was 5 Hz, and the spot size was 150 μm. Transient signals of selected isotopes (^{7}Li, ^{11}B, ^{13}C, ^{23}Na, ^{26}Mg, ^{27}AI, ^{29}Si, ^{31}P, ^{34}S, ^{35}Cl, ^{39}K, ^{43}Ca, ^{45}Sc, ^{49}Ti, ^{51}V, ^{53}Cr, ^{55}Mn, ^{57}Fe, ^{59}Co, ^{61}Ni, ^{65}Cu, ^{66}Zn, ^{71}Ga, ^{75}As, ^{77}Se, ^{79}Br, ^{85}Rb, ^{88}Sr, ^{89}Y, ^{90}Zr, ^{93}Nb, ^{95}Mo, ^{107}Ag, ^{111}Cd, ^{113}In, ^{118}Sn, ^{121}Sb, ^{133}Cs, ^{137}Ba, ^{139}La, ^{140}Ce, ^{141}Pr, ^{143}Nd, ^{149}Sm, ^{153}Eu, ^{155}Gd, ^{159}Tb, ^{161}Dy, ^{165}Ho, ^{166}Er, ^{169}Tm, ^{173}Yb, ^{175}Lu, ^{178}Hf, ^{182}W, ^{197}Au, ^{202}Hg, ^{208}Pb, ^{209}Bi, ^{232}Th, ^{238}U) were registered with 5 ms dwell time during a muti-line ablation over the area selected at the surface of the samples. Blank values, which were recorded for each selected isotope prior to the start of the ablation process, between each line ablation, and following the multi-line ablation, were subtracted from the signals obtained during the sample ablation.

Elemental distribution maps were generated by visualizing the variability of net signals. The total quantity of each element was estimated by standardizing each isotope to a 1% abundance level and by normalizing all observed isotopes at each data point to a total of 100%. Maps comprising 2855 individual data points were generated. The respective maps are shown with a common color scale. The scale of maps varies from white, indicating a detection limit equal to the mean blank signal plus three standard deviations for a given isotope, through grey at the

50th percentile level for a specific map, to red for signals at the 90th percentile height level. This method of data visualization allows for a rough estimation of the relative content of a specific element at a given location and demonstrates its distribution within the sample, enabling assessment of the homogeneity of its surface distribution.

Electrospinning procedure

The high voltage was connected to a stainless-steel needle (27 G) attached to the syringe with polymeric suspension. The flow of polymeric suspension was 1 ml/h, and its value was controlled by syringe pump (KDS100, Kd Scientific). Fiber mats were collected on an electrically grounded target of aluminum foil. The voltage applied during electrospinning nanofibers mat preparation was set at 12.5 kV, with distance between needle and collector equal to 10 cm. Electrospinning was carried at 21°C with humidity of 35% controlled by dehydrator and air conditioning. The nanofiber mats were collected for 3.8 h during electrospinning process.

Preparation of classical membranes K-ISE

K-ISE membranes, used in control experiments, contained (by weight): 1.4% KTFPB (or alternatively NaTFPB), 2.8% valinomycin, 64.2% DOS and 31.6% PVC. Total 100 mg were dissolved in 1 ml of THF. 2 ml of potassium selective cocktail was drop cast on Petri dish (diameter 4 cm) and left for solvent evaporation. Portion of this film, a square 0.8 cm x 0.8 cm, was used to prepare continuous film (classical) membranes sensors, used in parallel control experiments. The portion of the membrane was carefully placed on carbon fiber paper (below the opening) similarly as nanofibers mat and the whole system was sealed by lamination.

a) b)

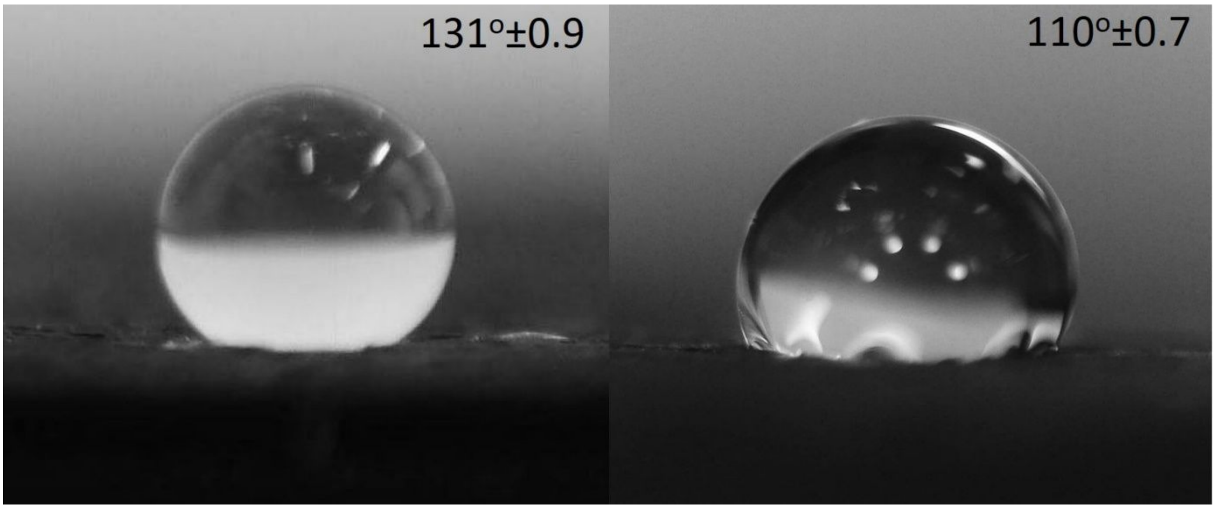

Fig. S1 Water contact angle determined for a) unmodified carbon paper support, b) carbon paper modified with DOS

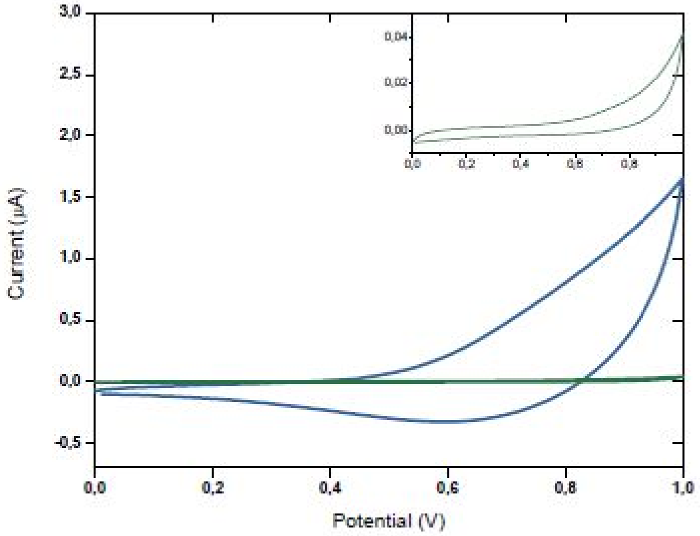

Fig. S2 Cyclic voltammograms of (green line) unmodified carbon paper and (blue line) carbon paper modified by application of 0.5 μL DOS plasticizer on carbon paper. Inset: magnified trace recorded for unmodified carbon paper. Scan rate 50 mV/s, 0.1 M KCl. For equivalent amount

of DOS drop cast on glassy carbon substrate, under the same experimental conditions, no redox responses were observed (results not shown).

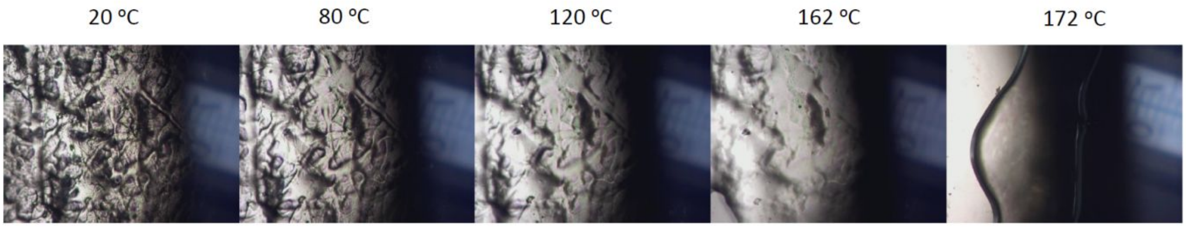

Fig. S3 Images of changes of the nanofibers mat structure during heating.

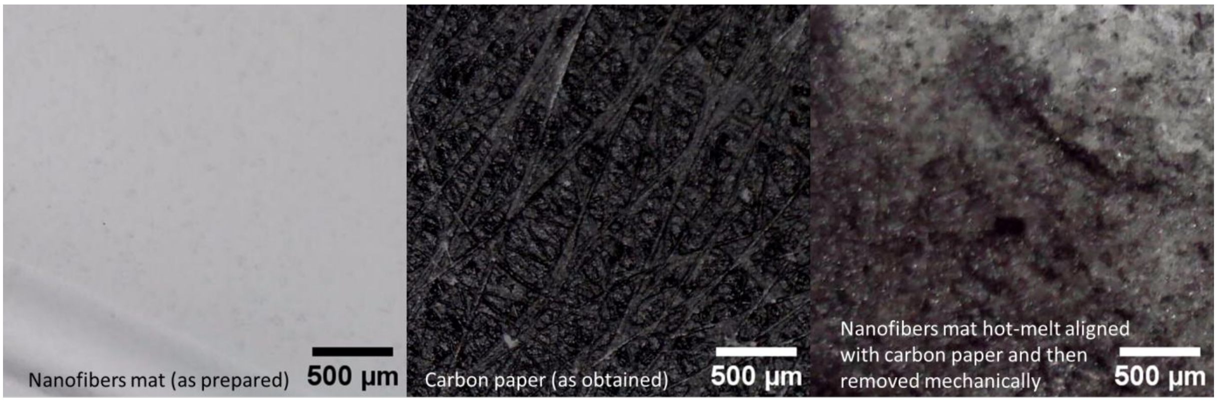

Fig. S4 Images of nanofibers mat (as prepared), carbon paper (as obtained) and nanofibers ISM removed post hot-melt process from carbon paper.

a) b)

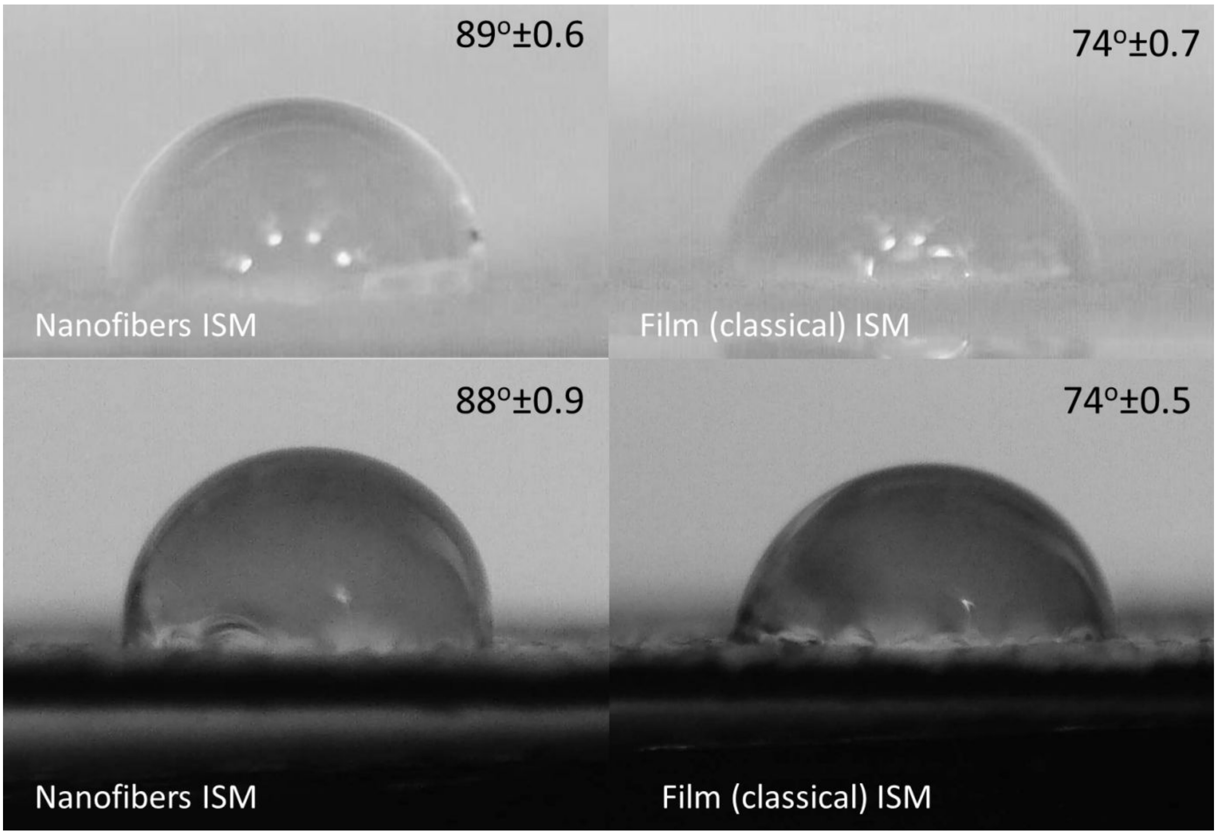

Fig. S5. Water contact angle determined for nanofibers ISM A) nanofibers, B) film membrane post hot-melt process and before the hot-melt process.

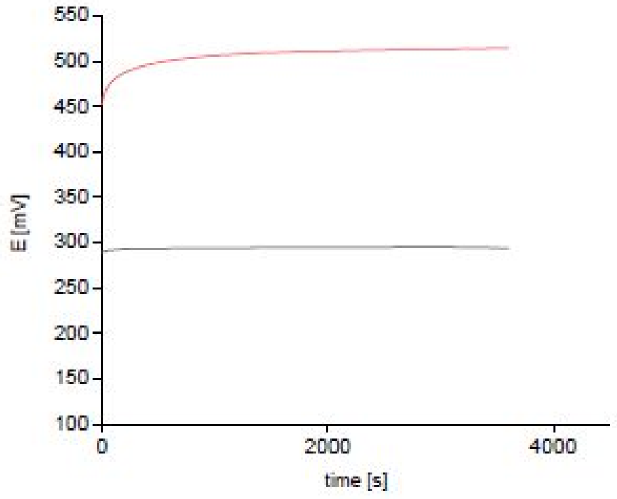

Fig S6 Equilibration time of the first contact of the sensor with 10^{-1} M KCl solution: (black line) nanofibers ISM sensor, (red line) classical (continuous film membrane) electrode.

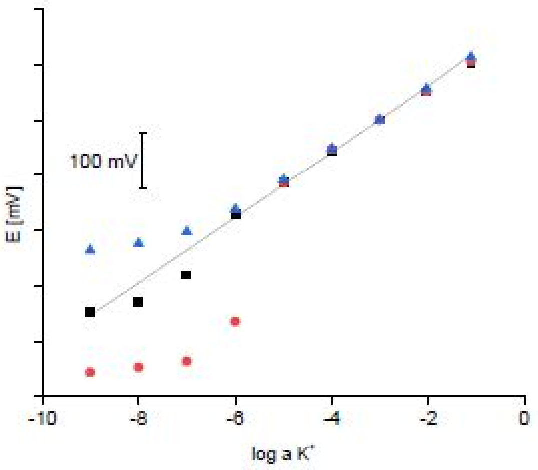

Fig. S7 Potentiometric responses of carbon coated potassium selective electrodes prepared using hot-melt process to nanofibers ISM and support, using NaTFPB ion-exchanger after conditioning for 40 minutes in 10^{-3} M KCl: (■) nanofibers mat or (●) continuous film membrane, recorded in KCl solutions, (▲) for comparison responses of sensors with nanofibers mat or reduced thickness (half of usual thickness, i.e. mate 20 µm thick) are also shown. Line represents Nernstian slope dependence.

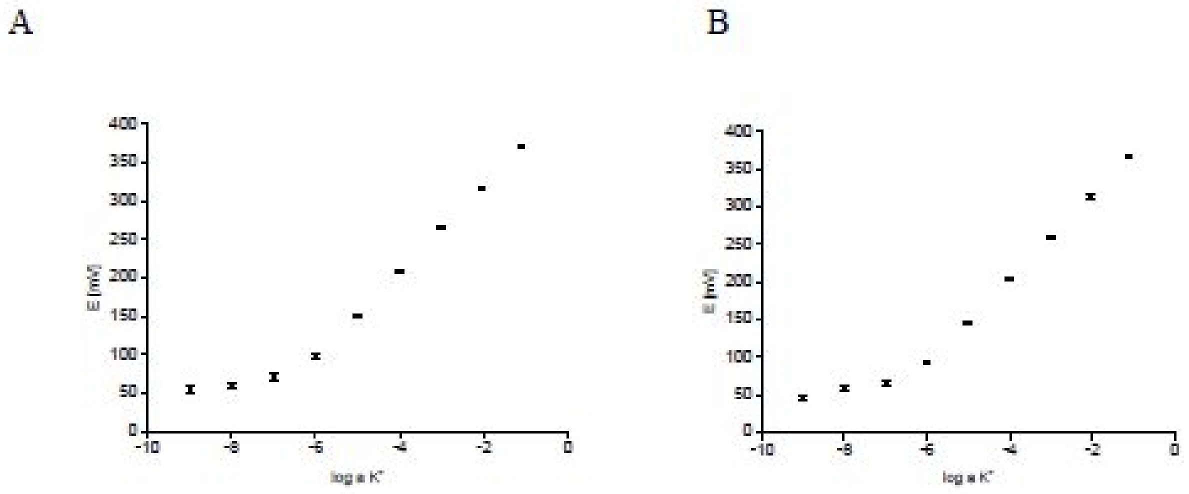

Fig. S8 Mean values of potentials ± SD obtained for A) one sensor from 8 calibrations and B) ten nominally identically sensors, tested in KCl solutions within the range from 10^{-9} to 10^{-1} M KCl.

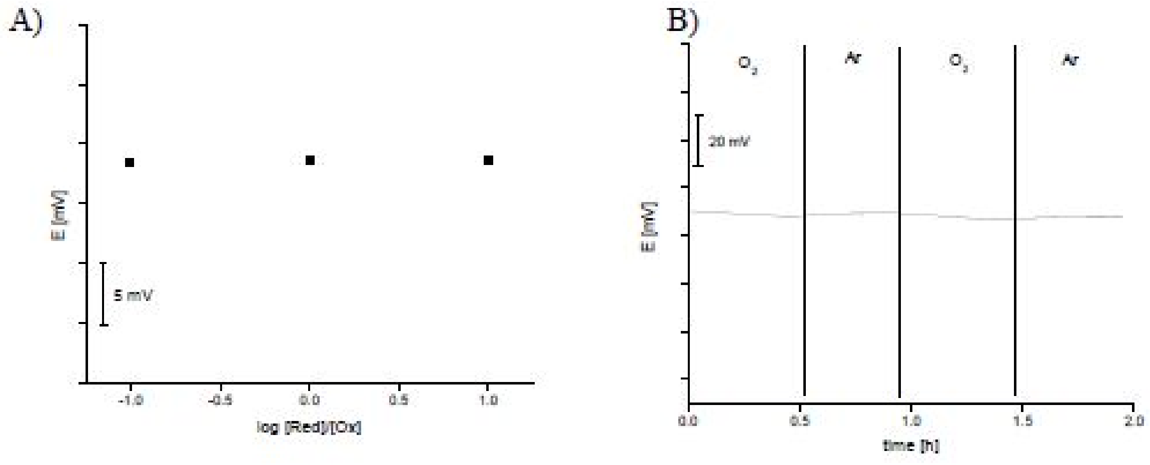

Fig. S9 Effect of redox reactants present in the solution on responses of nanofibers ISM based sensors: A) the dependence of EMF on logarithm of the ratio of reduced and oxidized components concentration of $Fe(CN)_6^{3-/4-}$ couple in the presence of KCl constant concentration 0.1 M; B) effect of O_2 / Ar presence in the 0.1 M KCl solution on the recorded potentials.

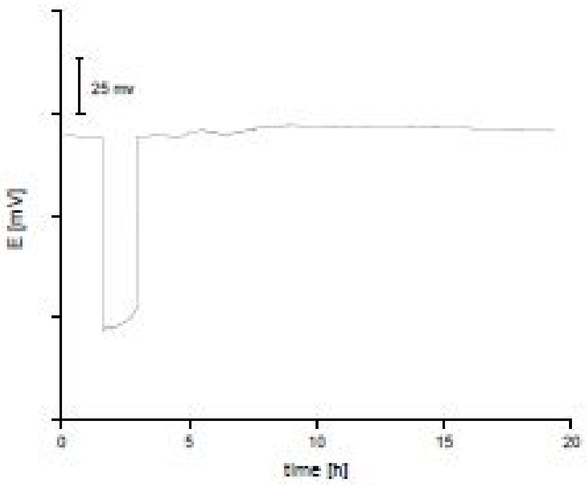

Fig. S10 Water later test results of nanofibers ISM based sensors, tested in 0.001 M KCl, 0.001 M NaCl and again in 0.001 M KCl.

A

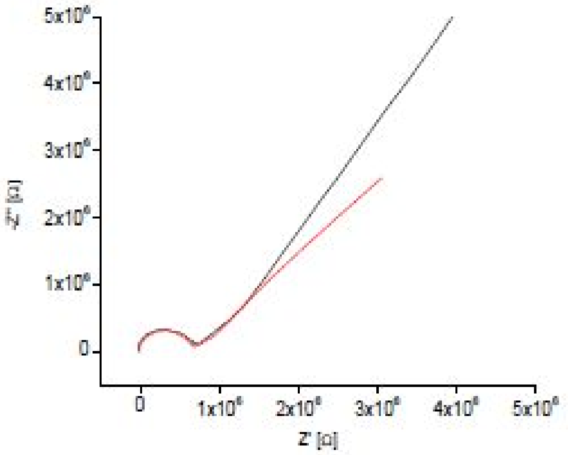

B

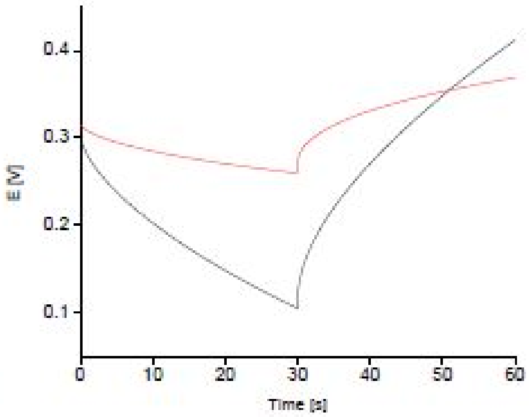

C

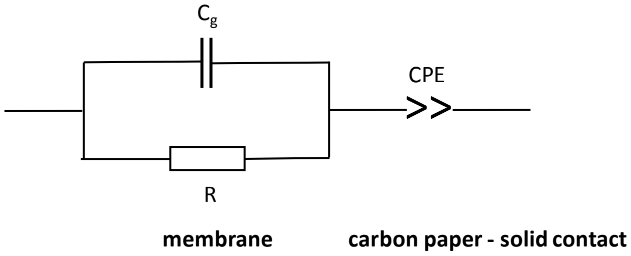

Fig. S11 Electrochemical tests results for (▬) nanofibers ISM based coated carbon paper sensors and sensors with (▬) classical (continuous) film sensors tested in parallel recorded in 0.1 M KCl: A) EIS spectra recorded at 0.3 V using amplitude 50 mV. B) Chronopotentiograms recorded applying 10^{-8} A cathodic/ anodic current. C) Equivalent circuit corresponding to EIS results from (A).

Table S1. Parameters of calibration lines obtained for tested sensors. Selectivity coefficients determined by separate solution method within range from 10^{-1} to 10^{-4} M, using experimental slopes.

	Nanofibers mat after 40 min. conditioning	Nanofibers mat after 20 h conditioning	Membrane after 40 min. conditioning
Lower detection limit (M)			
	$10^{-6.6}$	$10^{-6.7}$	$10^{-5.8}$
Slope ± SD, range, (R^2)			
	56.3 ± 0.2<br>10^{-1} - 10^{-6}<br>(0.999)	58.1 ± 0.5<br>10^{-1} - 10^{-6}<br>(0.999)	53.7 ± 1.4<br>10^{-1} - 10^{-5}<br>(0.998)
Logarithm of selectivity coefficient ($\log K_{I,J}^{pot}$ ± SD)			
Na^+	-3.6 ± 0.1	-3.3 ± 0.2	-3.4 ± 0.5
Mg^{2+}	-5.3 ± 0.2	-5.1 ± 0.3	-3.9 ± 0.2
Ca^{2+}	-3.5 ± 0.6	-3.2 ± 0.4	-3.4 ± 0.3
H^+	-4.5 ± 0.6	-4.3 ± 0.5	-5.1 ± 0.9
